# Duration of SARS-CoV-2 RNA positivity from various specimens and clinical characteristics in patients with COVID-19: a systematic review and meta-analysis

**DOI:** 10.1186/s41232-022-00205-x

**Published:** 2022-06-01

**Authors:** Yasutaka Okita, Takayoshi Morita, Atsushi Kumanogoh

**Affiliations:** 1 Department of Respiratory Medicine and Clinical Immunology, Osaka University Graduate School of Medicine, 2-2, Yamadaoka, Suita, Osaka 565-0871 Japan; 2 Department of Immunopathology, World Premier International Immunology Frontier Research Center (iFReC), Suita, Osaka Japan; 3 Integrated Frontier Research for Medical Science Division, Institute for Open and Transdisciplinary Research Initiatives (OTRI), Osaka University, Suita, Osaka Japan; 4 Center for Infectious Disease Education and Research (CiDER), Osaka University, Suita, Osaka Japan

**Keywords:** COVID-19, SARS-CoV-2, SARS-CoV-2 RNA positivity, Viral shedding, Coronavirus, Meta-analysis, Systematic review

## Abstract

**Background:**

The duration of severe acute respiratory syndrome coronavirus 2 (SARS-CoV-2) RNA positivity will be important to prevent the spread of coronavirus disease 2019 (COVID-19). A systematic review and meta-analysis were conducted following PRISMA to determine the duration from several parts of the body and clinical characteristics affecting it.

**Main text:**

PubMed, Web of Science, Scopus, and CENTRAL were searched for original studies reporting the duration from COVID-19 onset to the disappearance of viral RNA. Of the 1682 studies identified, 100 met the selection criteria and 13,431 patients were included in this study. The duration of SARS-CoV-2 RNA positivity was 18.29 [95% confidence interval: 17.00–19.89] days in the upper respiratory tract samples, 23.79 [20.43–27.16] days in the sputum, 14.60 [12.16–17.05] days in the blood, and 22.38 [18.40–26.35] days in the stool. Sensitivity analysis revealed that the duration was positively correlated with age, comorbidities, severity, and usage of glucocorticoid. Subgroup analysis indicated that the presence or absence of complications had the greatest impact on the difference in DSRP.

**Conclusions:**

The duration of SARS-CoV-2 RNA positivity was 18.29 days in the upper respiratory tract samples. The duration in the sputum and the stool was longer, while that in the blood was shorter. The duration in the upper respiratory tract samples was longer in older, with any comorbidities, severer, and treated with glucocorticoid. These results provide the basic data for the duration of SARS-CoV-2 RNA positivity, and in the future, the effect of vaccination against SARS-CoV-2 and the SARS-CoV-2 variants on the duration of RNA positivity should be assessed.

## Background

Coronavirus disease 2019 (COVID-19) is an infectious disease caused by severe acute respiratory syndrome coronavirus 2 (SARS-CoV-2). COVID-19 was first reported in China in December 2019 and became a pandemic [1]. Every country took infection control measures (e.g., lockdown), but the number of patients with COVID-19 increased worldwide. The quarantine period for COVID-19 varies from country to country. For example, the Centers for Disease Control and Prevention recommends 5 days for the general population [2]; the Ministry of Health, Labor and Welfare in Japan recommends 10 days from the onset [3]; and the China’s zero-COVID strategy recommends a longer period [4]. The result of reverse transcription-polymerase chain reaction (RT-PCR) is included in the de-quarantine criteria in Japan [3]. Detailed information on the duration of SARS-CoV-2 RNA positivity (DSRP) in various specimens of patients with COVID-19 will be very helpful in infection control.

SARS-CoV-2 RNA is detected in various samples such as nasal mucus, sputum, conjunctiva, blood, urine, gastric fluid, and stool [5]. It is certain that approximately 2 weeks after the onset was required for SARS-CoV-2 RNA to disappear from the respiratory tract in some studies [6, 7], but some cases were reported in which SARS-CoV-2 RNA had continued to be detected for a longer period [8]. The DSRP from other samples remains unclear due to the limited information. Moreover, whether the DSRP in patients with COVID-19 is affected by clinical characteristics remains unknown.

A systematic review of studies reporting the DSRP in patients with COVID-19 has been conducted and the DSRP from various specimens (nasal mucus, sputum, blood, and stool) was determined by a meta-analysis. Moreover, the influence of clinical features such as age, gender, comorbidity, severity, treatment, and locality on the DSRP was also evaluated for identification of the factors affecting the prolongation of DSRP.

## Methods

### Registration

This meta-analysis was performed following the Preferred Reporting Items for Systematic Reviews and Meta-analyses (PRISMA) statement [9] and registered with PROSPERO (CRD42020193268).

### Search strategy

Articles published until December 31, 2020, were searched for on PubMed, Web of Science, Scopus, and Cochrane CENTRAL using the search terms [(COVID-19 OR SARS-CoV-2) AND (shedding OR “viral load” OR clearance) AND patient NOT review] with no language restriction. The searches were performed thrice and the final searches were performed on February 15, 2021.

### Selection criteria

The inclusion criteria are studies of human subjects, original articles (not reviews), title or abstract consisting of the terms “COVID-19,” “SARS-CoV-2,” “shedding,” “viral load,” or “clearance,” and linkage to the full text of the article. Studies without raw data to calculate the mean and standard deviation (SD) of the DSRP were excluded. Case reports reporting one or two cases were excluded because it was difficult to calculate the mean and SD. Redundancies between the search sites were eliminated.

### Quality assessment

The quality assessment was performed following the study quality assessment tools (Quality Assessment Tool for Case Series Studies) from the National Heart, Lung, and Blood Institute (NHLBI) [10]. The evidence level was evaluated based on the Oxford Centre for Evidence-Based Medicine 2011 [11]. Funnel plots were used to assess publication bias.

### Data extraction

Author, year of publication, observational period, the country where the study was conducted, study design, number of patients, age, percentage of females, severity, treatment, comorbidity, and specimen were extracted. The severity was basically quoted from the severity classification used in each paper. In the studies not reporting it, the severity was classified according to the COVID-19 clinical classification released by the National Health Commission of China [12]. The DSRP was defined as the number of days from the appearance of symptoms to the first negative result of RT-PCR, not antigen test, without converting positive thereafter. The Ct (threshold cycle) value to be judged negative was quoted from the criteria used in each paper. The mean and SD of DSRP were extracted. In the studies reporting only the median and interquartile range (IQR) or range of DSRP, the mean and SD were calculated from them using the methods of Wan et al. [13]. Patients whose RT-PCR result for SARS-CoV-2 did not turn negative during the observation period were excluded. Asymptomatic patients were excluded because defining the onset was difficult. The values were manually calculated using information available in the published graphs and tables when raw data were unavailable.

### Statistics

In the meta-analysis, the DSRP were expressed as the mean number of days and 95% confidence intervals (CIs). The mean differences were calculated using the random effects model. *I*^2^ values of 25%, 50%, and 75% were defined as low, moderate, and high, respectively [14]. The sensitivity analyses were performed based on age, gender, comorbidities, compromised status, severity, and use of glucocorticoid. Spearman’s correlation coefficient was calculated and *p* values ≤0.05 were considered statistically significant. The subgroup analyses were performed between the patients with different ages, the patients with or without any comorbidities, the patients with different severities, the patients treated with and without glucocorticoid, and the studies from different countries. All analyses were conducted using the R version 4.0.0 (R Project for Statistical Computing) and EZR version 1.42 [15].

## Results

### Study selection

**Fig. 1:**
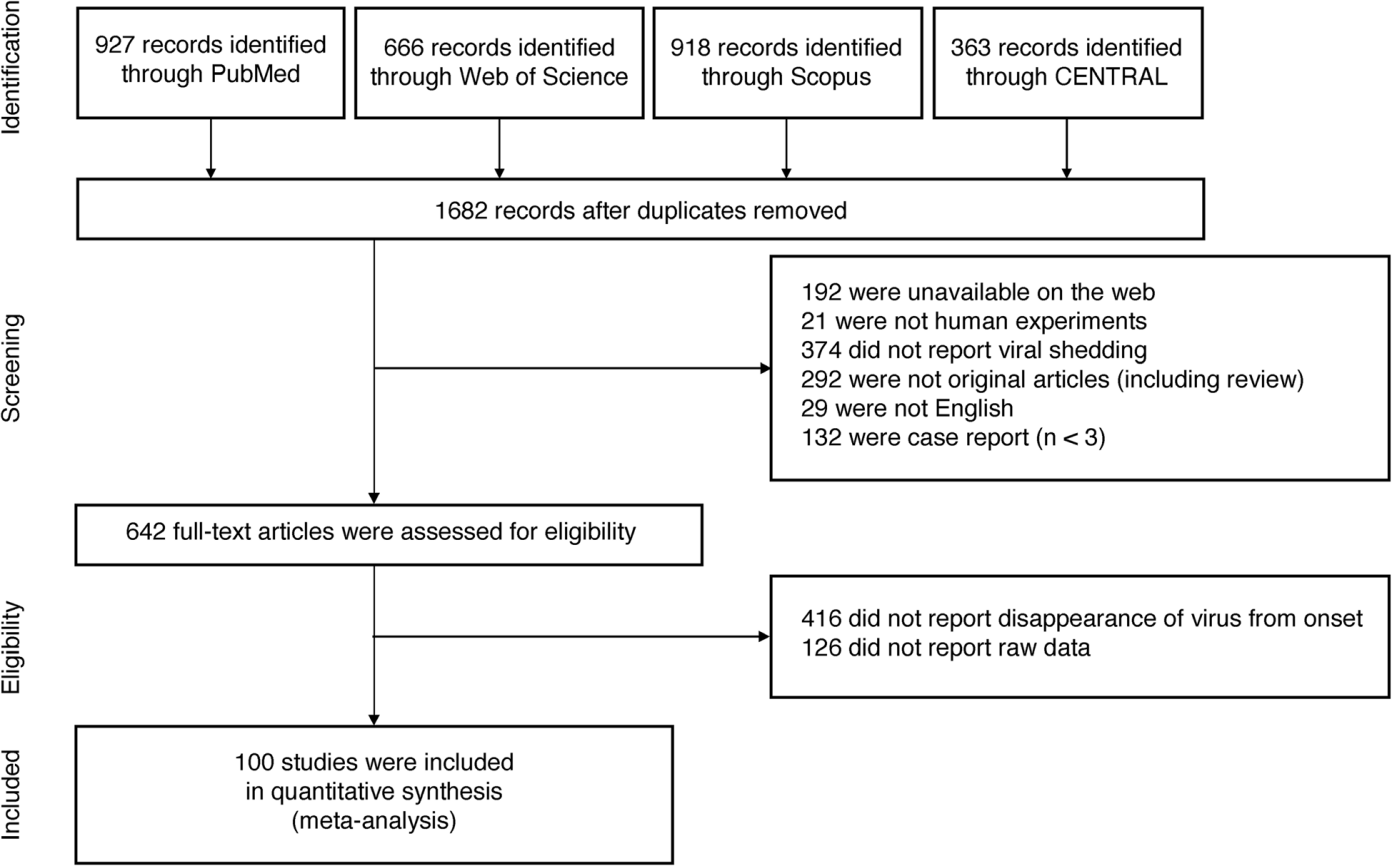
PRISMA flow diagram

The current study identified 1682 records from four search sites (927, 666, 918, and 363 studies on PubMed, Web of Science, Scopus, and Cochrane Central Register of Controlled Trials (CENTRAL), respectively). One thousand forty studies which did not meet the inclusion criteria were removed and 542 studies were removed based on the exclusion criteria. Finally, 100 studies met the selection criteria and were included in this meta-analysis (Fig. 1, [5–8, 16–111]).

### The characteristics of the studies, clinical characteristics, and quality assessment

Most studies were observational studies and were classified as case accumulation research from the viewpoint of the current study. Seventy-two, 15, and 13 studies were reported from China, Asian countries except for China, and European countries, respectively. The start of the observation period was December 29, 2019, to April 30, 2020, and the end was January 11, 2020, to June 10, 2020. The number of patients ranged from 3 to 1320 and the total number of patients with COVID-19 in the 100 studies was 13,431. The median age ranged from 6 to 74.5, with a minimum age of 0 to 49 years and a maximum age of 11 to 96 years. The proportion of women was 0 to 100%. The proportion of patients with any comorbidities was 6.3 to 100%. The proportion of severe patients ranged from 0 to 100%. The proportion of patients treated for COVID-19 with glucocorticoid ranged from 0 to 100%.

**Fig. 2:**
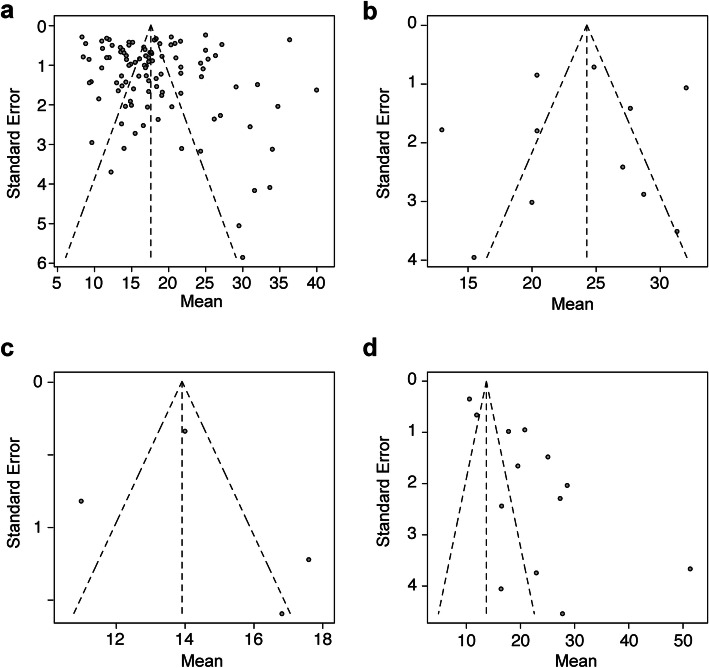
The funnel plots of the duration of SARS-CoV-2 RNA positivity in various samples. The funnel plots of the duration of SARS-CoV-2 RNA positivity in the upper respiratory tract samples (**a**), the sputum (**b**), the blood (**c**), and the stool (**d**) were shown

The total score of the study quality assessment tools (Quality Assessment Tool for Case Series Studies) from the NHLBI was in the range of 6 to 9 in each study (data not shown). The funnel plots in the upper respiratory tract samples including nasal swab and throat swab (Fig. 2a), sputum (Fig. 2b), blood (Fig. 2c), and stool (Fig. 2d) had asymmetrical isosceles, suggesting the presence of bias or systemic heterogeneity.

### Duration of SARS-CoV-2 RNA positivity on various respiratory tract samples

**Fig. 3:**
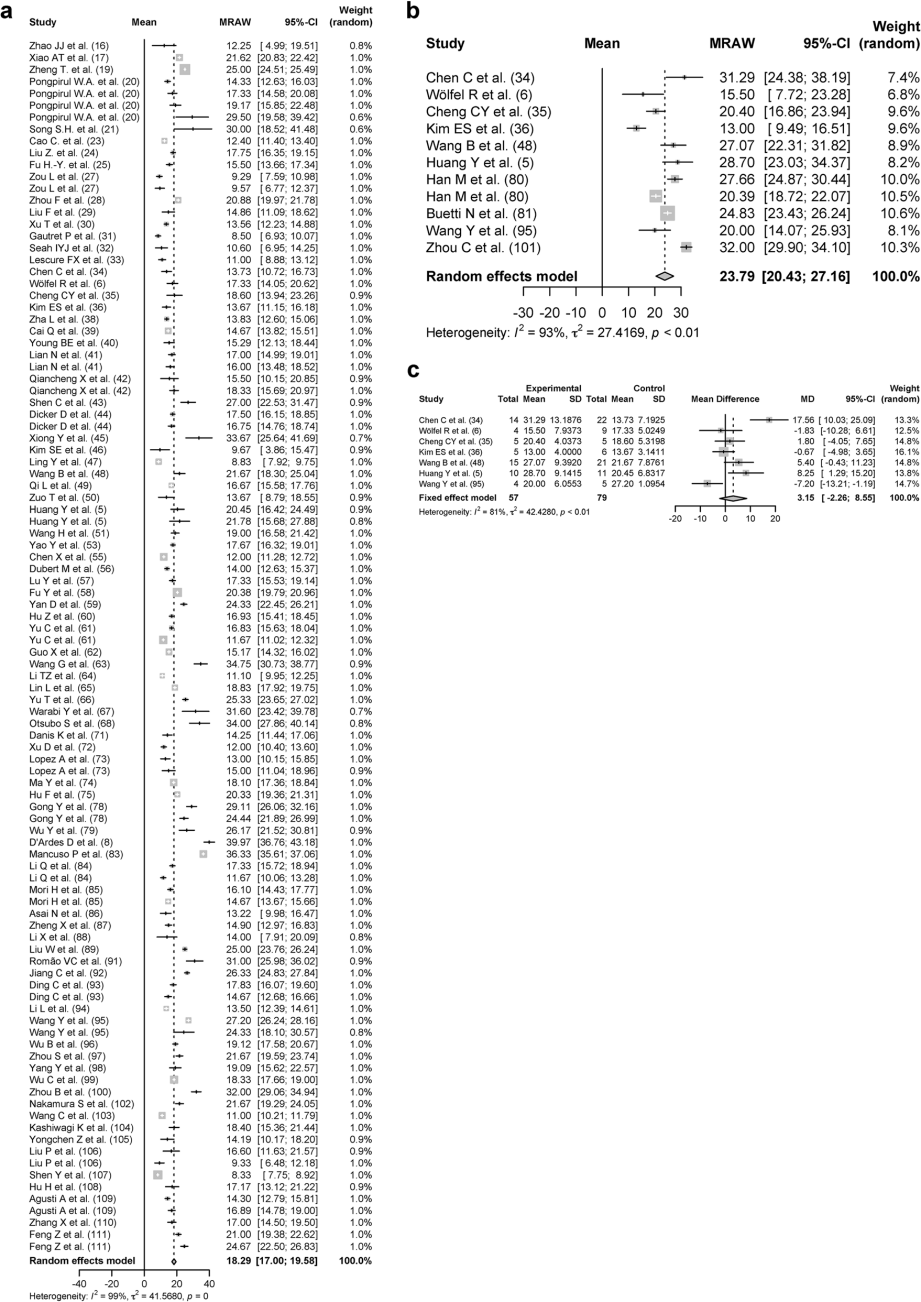
Forest plot: a meta-analysis of the duration of SARS-CoV-2 RNA positivity in the respiratory tract samples. The duration of SARS-CoV-2 RNA positivity from the onset of COVID-19 in the upper respiratory tract samples (**a**) and the sputum (**b**) was calculated using the random effects model. The difference in the duration of SARS-CoV-2 RNA positivity between the sputum and the upper respiratory samples was calculated using the random effects model (**c**). Experimental meant the sputum and control meant the upper respiratory tract samples. MRAW, the raw data of mean; 95%-CI, 95% confidence interval; SD, standard deviation; MD, mean difference

In all respiratory tract samples including nasal swab, throat swab, sputum, and bronchoalveolar lavage fluid, 11,639 patients from 99 studies were analyzed [5–8, 16–36, 38–111] with a DSRP of 18.79 days (95% CIs, 17.69–19.89 days, *I*^2^ = 99%). In the upper respiratory tract samples including nasal swab and throat swab, 9635 patients from 84 studies were analyzed with a DSRP of 18.29 days (95% CIs, 17.00–19.58 days, *I*^*2*^ = 99%; Fig. 3a). In the nasal swabs, 4042 patients from 32 studies were analyzed with a DSRP of 19.34 days (95% CIs, 16.60–22.07 days, *I*^*2*^ = 99%). In the throat swabs, 4631 patients from 44 studies were analyzed with a DSRP of 17.85 days (95% CIs, 16.43–19.26 days, *I*^*2*^ = 98%). In the sputum, 643 patients from 10 studies were analyzed with a DSRP of 23.79 days (95% CIs, 20.43–27.16 days, *I*^*2*^ = 93%; Fig. 3b). The DSRP on upper respiratory tract samples and sputum of 79 and 57 patients, respectively, were directly compared. The DSRP in the sputum tended to be 3.15 days longer (95% CIs, − 2.26–8.55 days, *p* < 0. 01, *I*^*2*^ = 81%; Fig. 3c) than the upper respiratory tract samples, but there was no significant difference.

### Duration of SARS-CoV-2 RNA positivity on samples from blood and stool

**Fig. 4:**
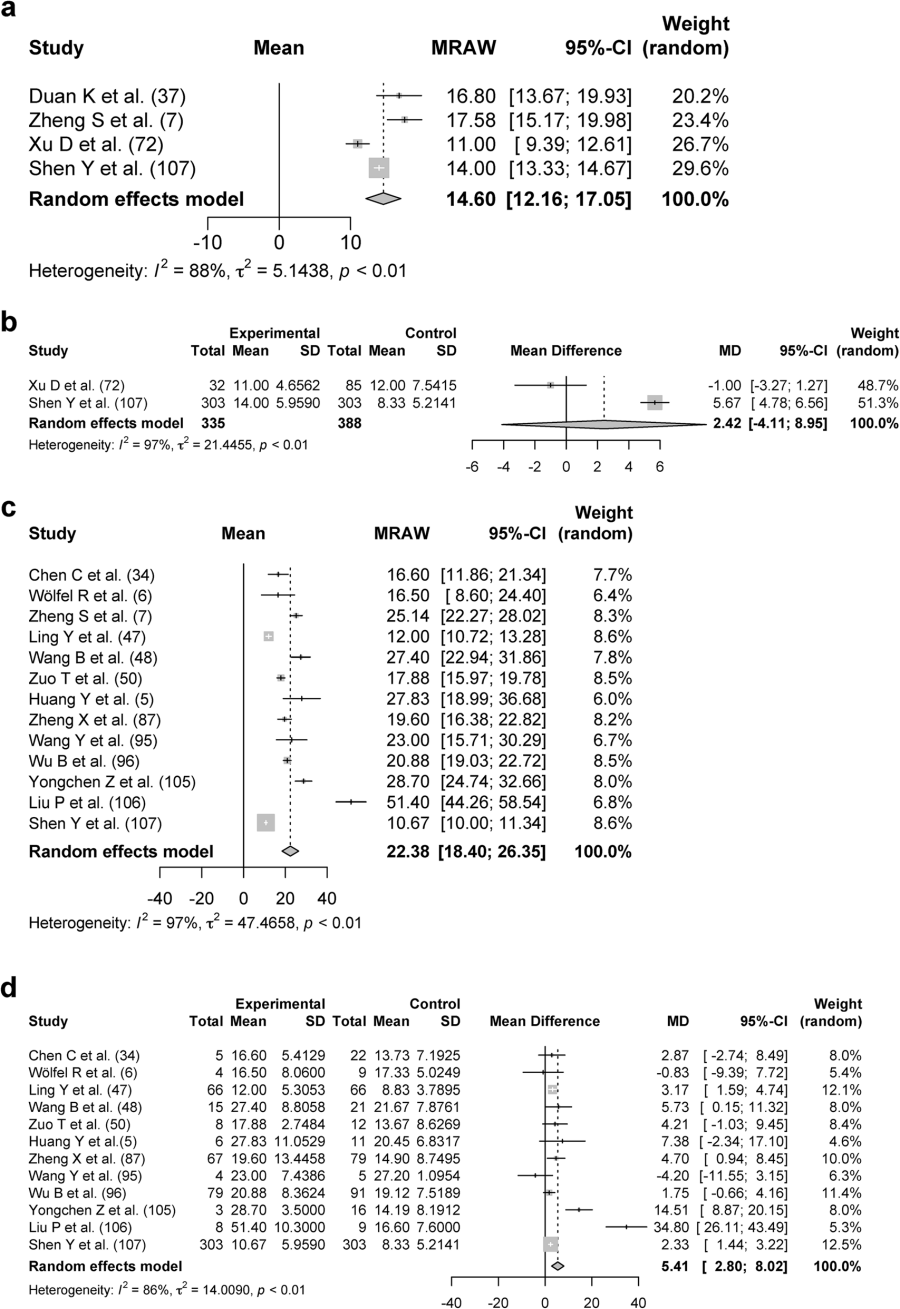
Forest plot: a meta-analysis of the duration of SARS-CoV-2 RNA positivity in various samples. The duration of SARS-CoV-2 RNA positivity from the onset of COVID-19 in the blood (**a**) and stool (**c**) was calculated using the random effects model. The difference in the duration of SARS-CoV-2 RNA positivity between the blood and upper respiratory tract samples was calculated using the random effects model (**b**). Experimental meant the blood samples and control meant the upper respiratory tract samples. The difference in the duration of SARS-CoV-2 RNA positivity between the stool and upper respiratory tract samples was calculated using the random effects model (**d**). Experimental meant the stool and control meant the upper respiratory tract samples. MRAW, the raw data of mean; 95%-CI, 95% confidence interval; SD, standard deviation; MD, mean difference

In the blood, 385 patients from four studies were analyzed with a DSRP of 14.60 days (95% CIs, 12.16–17.05 days, *I*^*2*^ = 88%; Fig. 4a). The DSRP on the blood and the upper respiratory tract samples from 335 and 388 patients, respectively, were directly compared, and there was no significant difference (2.42 days; 95% CIs − 4.11–8.95 days, *p* < 0.01, *I*^*2*^ = 97%; Fig. 4b). In the stool, 620 patients from 13 studies were analyzed with a DSRP of 22.38 days (95% CIs, 18.40–26.35 days, *I*^*2*^ = 97%; Fig. 4c). The DSRP on the stool and the upper respiratory tract samples from 568 and 644 patients, respectively, were directly compared. The DSRP on the stool was significantly 5.41 days longer (95% CIs, 2.80–8.02 days, *p* < 0.01, *I*^*2*^ = 86%) than the upper respiratory tract samples (Fig. 4d).

### Sensitivity analysis based on the clinical characteristics in upper respiratory tract samples

**Fig. 5:**
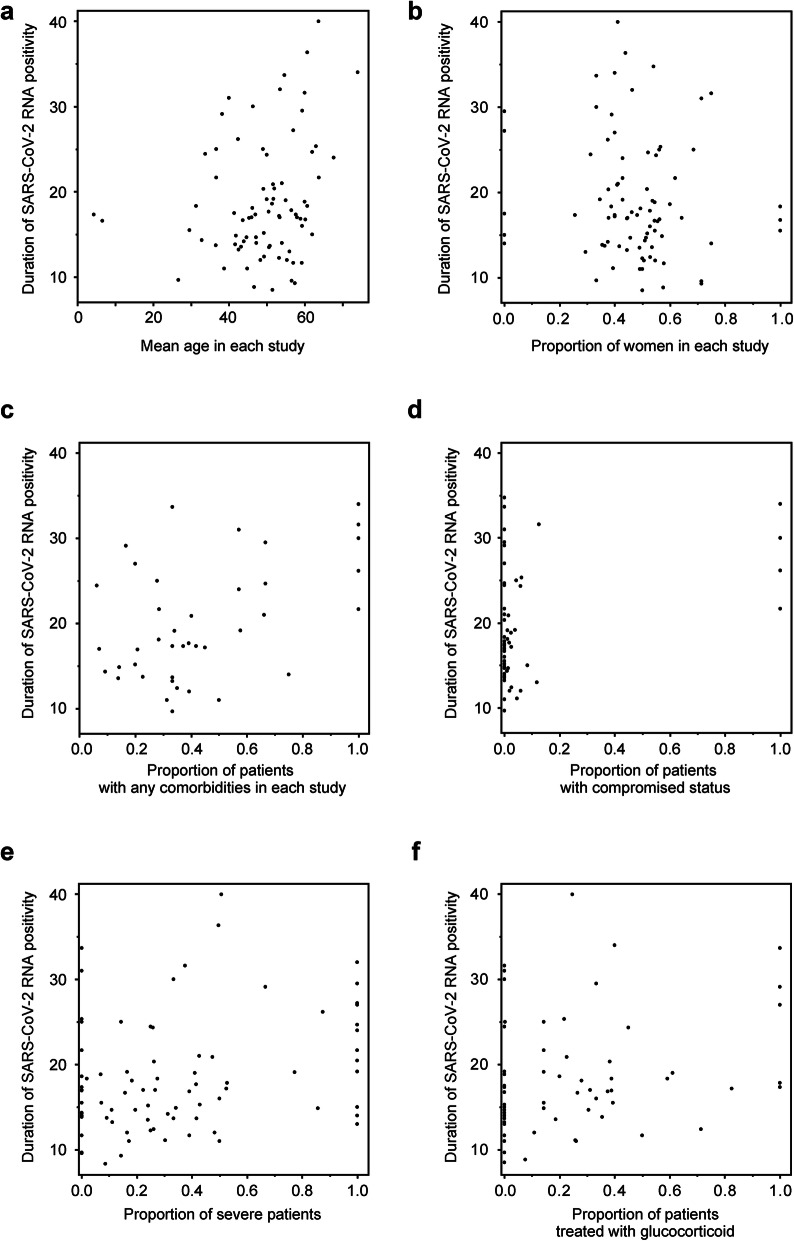
Sensitivity analysis based on the clinical characteristics in upper respiratory tract samples. The association between the duration of SARS-CoV-2 RNA positivity from the onset of COVID-19 in upper respiratory tract samples and mean age (**a**), the proportion of women (**b**), the proportion of patients with any comorbidities (**c**), the proportion of patients with compromised status (**d**), the proportion of severe patients (**e**), and the proportion of patients treated with glucocorticoid (**f**) in each study. The correlation was evaluated using the Spearman correlation coefficient

In the upper respiratory tract samples, sensitivity analyses were performed. The mean age was significantly positively correlated with the DSRP (*ρ* = 0.22, *p* = 0.05; Fig. 5a), while the proportion of women was not (*ρ* = − 0.14, *p* = 0.19; Fig. 5b). The proportion of patients with any comorbidities was significantly positively correlated with the DSRP (*ρ* = 0.35, *p* = 0.02; Fig. 5c), while the proportion of patients with compromised status such as malignancy, human immunodeficiency virus infection, and dialysis treatment was not (*ρ* = 0.14, *p* = 0.32; Fig. 5d). The proportion of severe patients was significantly positively correlated with the DSRP (*ρ* = 0.26, *p* = 0.02; Fig. 5e), and the proportion of patients treated with glucocorticoid was significantly positively correlated with the DSRP (*ρ* = 0.26, *p* = 0.04; Fig. 5f). It was suggested that the age, comorbidities, severity, and usage of glucocorticoid affected the DSRP, and the percentage of patients with any comorbidities had the greatest impact on DSRP based on the value of *ρ*.

### Subgroup analysis based on the age, comorbidities, severity, and usage of glucocorticoid in the upper respiratory tract samples

**Fig. 6:**
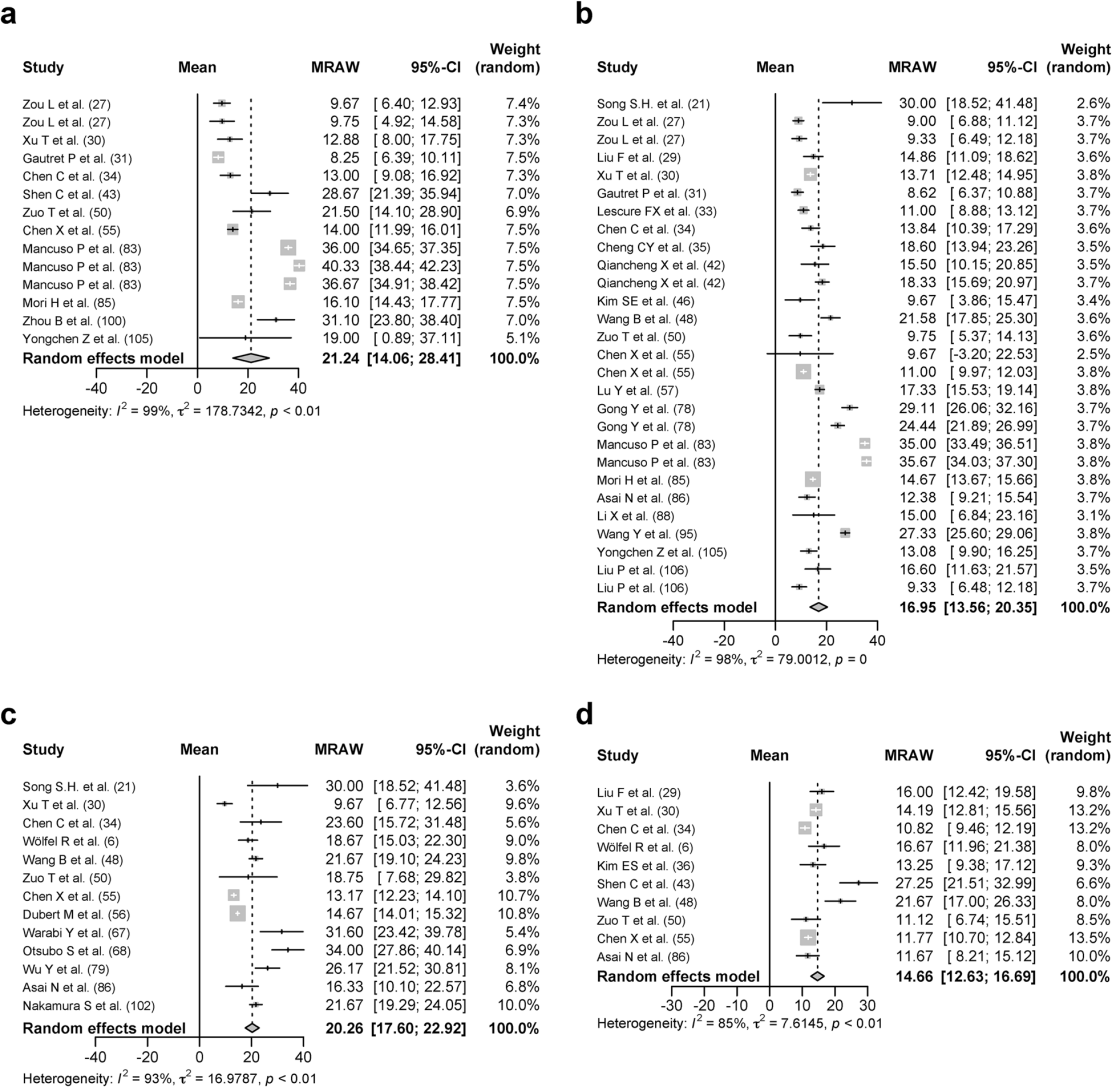
Forest plot: a meta-analysis of the duration of SARS-CoV-2 RNA positivity in the upper respiratory tract sample based on age and comorbidities. The duration of SARS-CoV-2 RNA positivity from the onset of COVID-19 in the patients over the age of 60 (**a**), patients under the age of 60 (**b**), patients with any comorbidities (**c**), patients without any comorbidities patients (**d**) was calculated using the random effects model. MRAW, the raw data of mean; 95%-CI, 95% confidence interval

Seven hundred forty-two patients over the age of 60 (older group) from 11 studies were analyzed with a DSRP of 21.24 days (95% CIs, 14.06–28.41 days, *I*^*2*^ = 99%; Fig. 6a). One thousand one hundred twenty-nine patients under the age of 60 (younger group) from 22 studies were analyzed with a DSRP of 16.95 days (95% CIs, 13.56–20.35 days, *I*^*2*^ = 98%; Fig. 6b). The mean age was 68.03 ± 3.12 years in the older group and 36.41 ± 12.05 years in the younger group. The proportion of patients with any comorbidities was 44.79 ± 20.23% in the older group and 28.06 ± 26.85% in the younger group. The proportion of severe patients was 61.90 ± 40.50% in the older group and 22.27 ± 31.21% in the younger group. The proportion of patients treated with glucocorticoid was 37.50 ± 47.87% in the older group and 13.26 ± 26.48% in the younger group. Due to many missing data values, the number of patients in the older group was less than 30 after further adjustment of the patient background. It was judged that the analysis would not be appropriate.

One hundred eighty patients with any comorbidities (comorbidity group) from 13 studies were analyzed with a DSRP of 20.26 days (95% CIs, 17.60–22.92 days, *I*^2^ = 93%; Fig. 6c). Two hundred sixty-five patients without any comorbidities (noncomorbidity group) from 10 studies were analyzed with a DSRP of 14.66 days (95% CIs, 12.63–16.69 days, *I*^2^ = 85%; Fig. 6d). The mean age was 57.10 ± 8.94 years in the comorbidity group and 37.88 ± 5.76 years in the noncomorbidity group. The proportion of severe patients was 46.67 ± 37.75% in the comorbidity group and 36.55 ± 44.59% in the noncomorbidity group. The proportion of patients treated with glucocorticoid was 8.87 ± 14.41% in the comorbidity group and 27.11 ± 41.71% in the noncomorbidity group. Due to many missing data values, the numbers of patients in both groups were less than 30 after further adjustment of the patient background. It was judged that the analysis would not be appropriate.

**Fig. 7:**
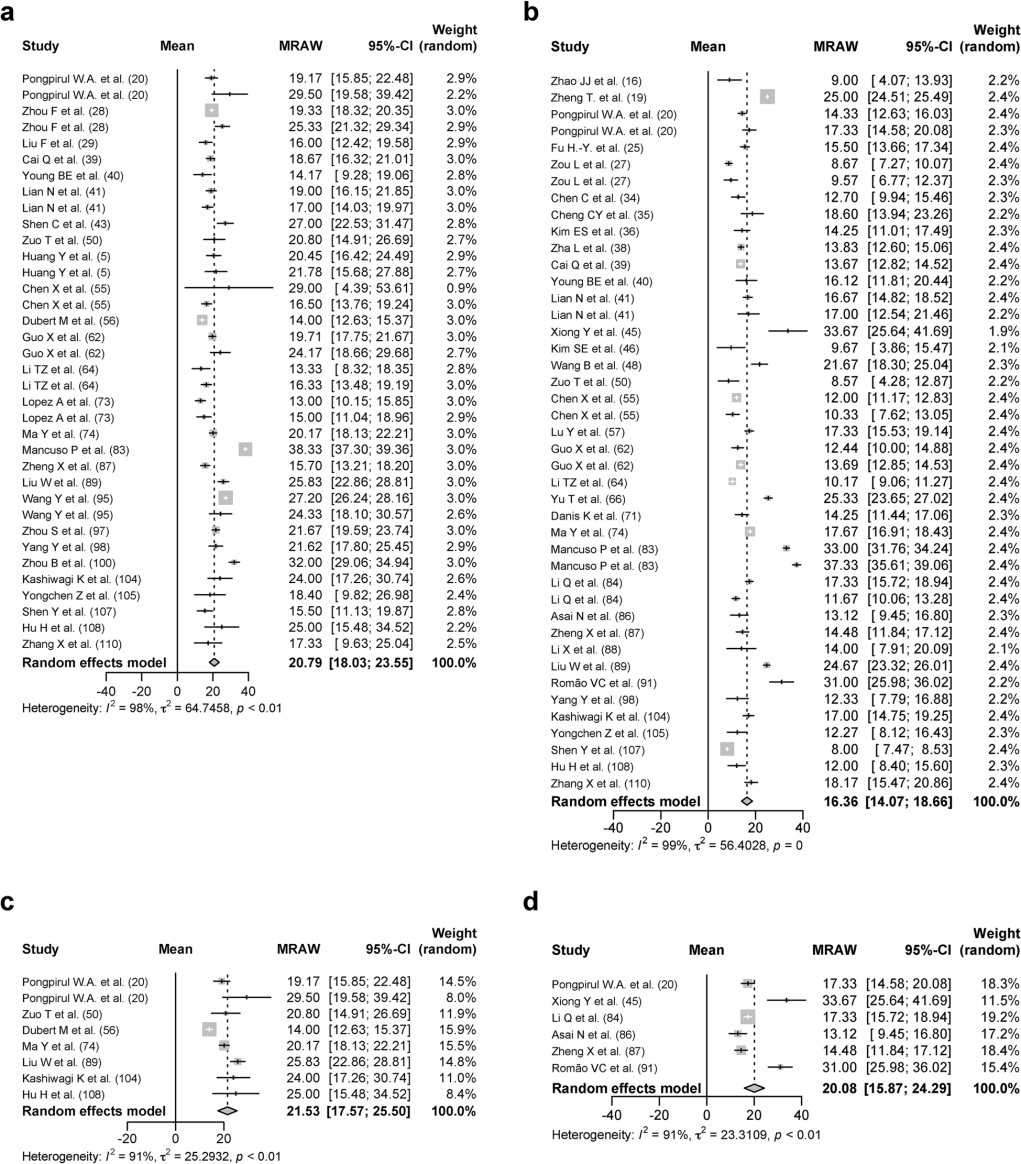
Forest plot: a meta-analysis of the duration of SARS-CoV-2 RNA positivity in the upper respiratory tract samples based on the severity. The duration of SARS-CoV-2 RNA positivity from the onset of COVID-19 in the severe patients (**a**) and the nonsevere patients (**b**) was calculated using the random effects model. The duration of SARS-CoV-2 RNA positivity from the onset of COVID-19 in the severe patients (**c**) and the nonsevere patients (**d**) from the studies with the mean age of 40 years or older and the proportion of patients with any comorbidities of 30% or more was calculated using the random effects model. MRAW, the raw data of mean; 95%-CI, 95% confidence interval

One thousand three hundred thirty-nine severe patients from 27 studies were analyzed with a DSRP of 20.79 days (95% CIs, 18.03–23.55 days, *I*^2^ = 98%; Fig. 7a). Four thousand two hundred nineteen nonsevere patients from 36 studies were analyzed with a DSRP of 16.36 days (95% CIs, 14.07–18.66 days, *I*^2^ = 99%; Fig. 7b). The mean age was 57.16 ± 6.01 in the severe patients and 44.12 ± 11.17 years in the nonsevere patients. The proportion of patients with any comorbidities was 51.05 ± 28.73% in the severe patients and 28.15 ± 12.91% in the nonsevere patients. The proportion of patients treated with glucocorticoid was 21.74 ± 39.91% in the severe patients and 20.43 ± 31.49% in the nonsevere patients. To adjust those factors as further as possible between the severe patients and the nonsevere patients, studies with the mean age of 40 years or older and the proportion of patients with any comorbidities of 30% or more were selected. One hundred seventy-one severe patients were analyzed with a DSRP of 21.53 days (95% CIs 17.57–25.50 days, *p* < 0.01, *I*^*2*^ = 91%; Fig. 7c). One hundred seventy-five nonsevere patients were analyzed with a DSRP of 20.08 days (95% CIs 15.87–24.29 days, *p* < 0.01, *I*^*2*^ = 91%; Fig. 7d). It was suggested that the severity of COVID-19 had a mild effect on the DSRP.

**Fig. 8:**
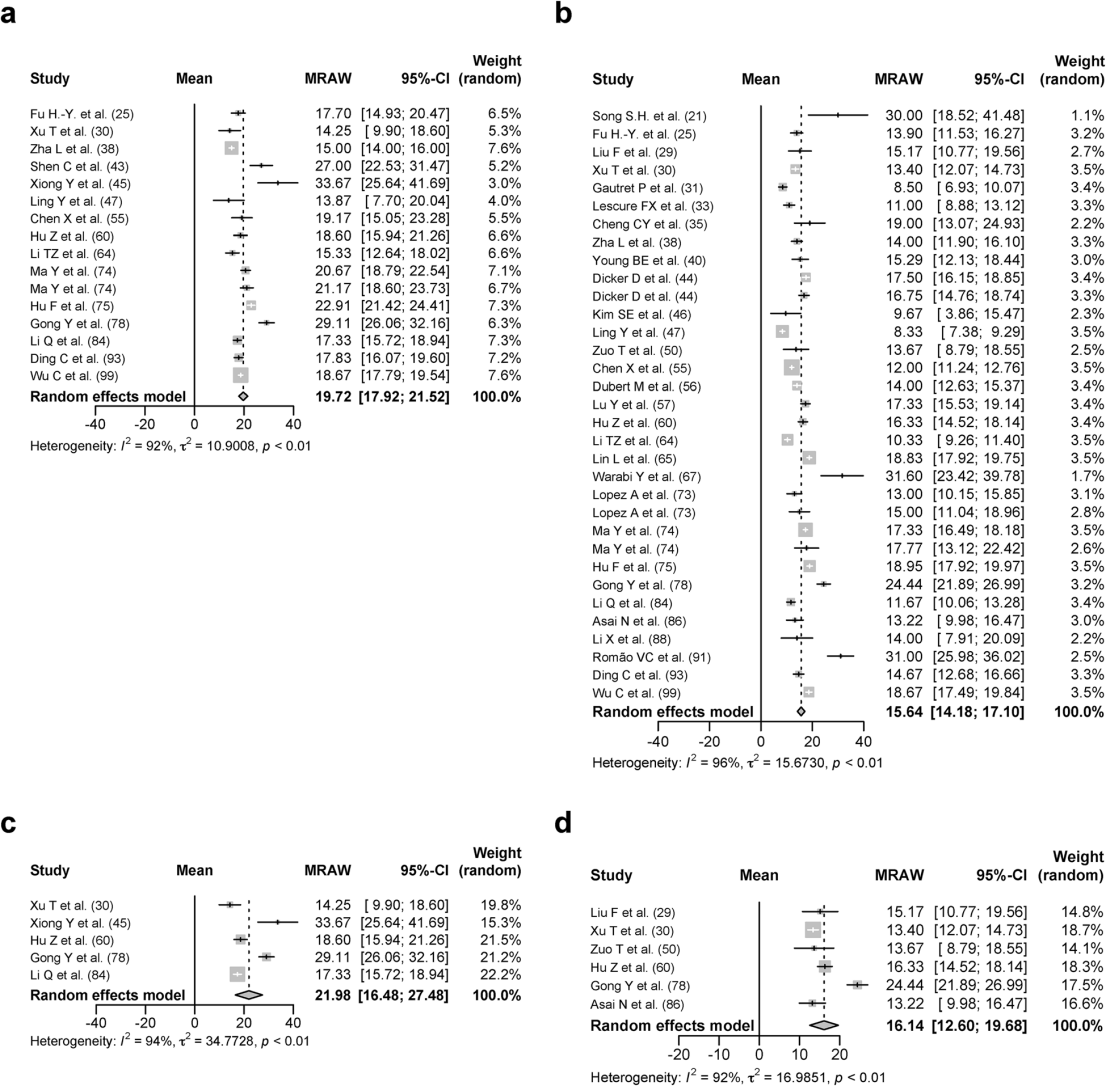
Forest plot: a meta-analysis of the duration of SARS-CoV-2 RNA positivity in the upper respiratory tract samples based on the usage of glucocorticoid. The duration of SARS-CoV-2 RNA positivity from the onset of COVID-19 in the patients treated with glucocorticoid (**a**) and the patients treated without glucocorticoid (**b**) was calculated using the random effects model. The duration of SARS-CoV-2 RNA positivity from the onset of COVID-19 in the patients treated with glucocorticoid (c) and the patients treated without glucocorticoid (**d**) from the studies with the mean age of 30–60 years and the proportion of patients with any comorbidities of 50% or less was calculated using the random effects model. MRAW, the raw data of mean; 95%-CI, 95% confidence interval

Six hundred forty patients treated with glucocorticoid (glucocorticoid group) from 15 studies were analyzed with a DSRP of 19.72 days (95% CIs, 17.92–21.52 days, *I*^2^ = 92%; Fig. 8a). One thousand six hundred seventy patients treated without glucocorticoid (no glucocorticoid group) from 30 studies were analyzed with a DSRP of 15.64 days (95% CIs, 14.18–17.10 days, *I*^2^ = 96%; Fig. 8b). The mean age was 52.64 ± 6.28 years in the glucocorticoid group and 46.25 ± 12.68 years in the no glucocorticoid group. The proportion of patients with any comorbidities was 24.89 ± 10.98% in the glucocorticoid group and 45.27 ± 31.88% in the no glucocorticoid group. The proportion of severe patients was 34.91 ± 42.06% in the glucocorticoid group and 31.95 ± 37.61% in the no glucocorticoid group. To adjust those factors as further as possible between the glucocorticoid group and the no glucocorticoid group, studies with the mean age of 30–60 years and the proportion of patients with any comorbidities of 50% or less were selected. One hundred twelve patients treated with glucocorticoid were analyzed with a DSRP of 21.98 days (95% CIs 16.48–27.48 days, *p* < 0.01, *I*^*2*^ = 94%; Fig. 8c). One hundred twenty-two patients treated without glucocorticoid were analyzed with a DSRP of 16.14 days (95% CIs 12.60–19.68 days, *p* < 0.01, *I*^*2*^ = 92%; Fig. 8d). It was suggested that the usage of glucocorticoid had a mild effect on the DSRP.

### Subgroup analysis based on locality in the upper respiratory tract samples

**Fig. 9:**
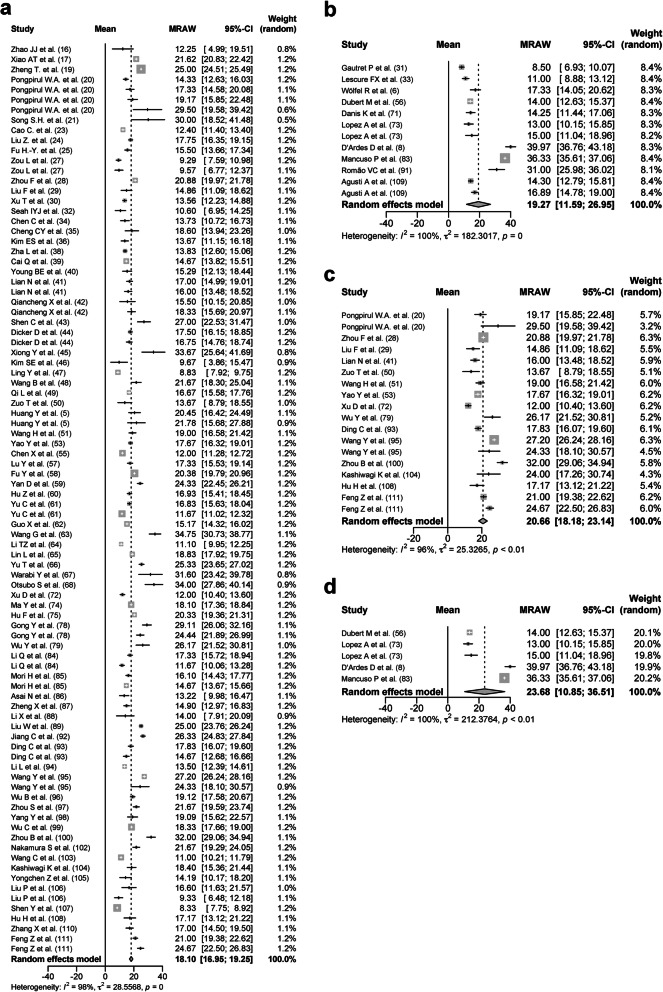
Forest plot: subgroup meta-analysis of the duration of SARS-CoV-2 RNA positivity from the onset of COVID-19 in the upper respiratory tract samples based on the locality. The duration of SARS-CoV-2 RNA positivity from the onset of COVID-19 in the Asian countries (**a**) and European countries (**b**) was calculated using the random effects model. The duration of SARS-CoV-2 RNA positivity from the onset of COVID-19 in the Asian countries (**c**) and European countries (**d**) from the studies with the mean age of 40 years or older and the proportion of severe patients of 40% or more was calculated using the random effects model. MRAW, the raw data of mean; 95%-CI, 95% confidence interval

In the upper respiratory tract samples, 8201 patients in Asian countries were analyzed with a DSRP of 18.10 days (95% CIs 16.95–19.25 days, *p* = 0, *I*^*2*^ = 98%; Fig. 9a). A total of 1434 patients in European countries were analyzed with a DSRP of 19.27 days (95% CIs 11.59–26.95 days, *p* = 0, *I*^*2*^ = 100%; Fig. 9b). The mean age was 48.61 ± 11.64 and 53.32 ± 9.54 years in Asian and European countries, respectively. The proportion of patients with any comorbidities was 42.74 ± 27.86% and 53.87 ± 17.26% in Asian and European countries, respectively. The proportion of severe patients was 33.75 ± 32.69% and 56.29 ± 41.72% in Asian and European countries, respectively. The proportion of patients treated with glucocorticoid was 28.09 ± 30.56% and 3.52 ± 9.31% in Asian and European countries, respectively. In studies from Asian countries, the patients were younger, the incidence of comorbidities was low, and COVID-19 was milder. However, glucocorticoid was used more in Asian countries. To adjust those factors as further as possible between Asian and European countries, studies with the mean age of 40 years or older and the proportion of severe patients of 40% or more were selected. Eight hundred thirty-one patients in Asian countries were analyzed with a DSRP of 20.66 days (95% CIs 18.18–23.14 days, *p* < 0.01, *I*^*2*^ = 96%; Fig. 9c). A total of 1268 patients in European countries were analyzed with a DSRP of 23.68 days (95% CIs 10.85–36.51 days, *p* < 0.01, *I*^*2*^ = 100%; Fig. 9d). It was suggested that the DSRP may be longer in patients in European countries.

### Summary out results

**Fig. 10:**
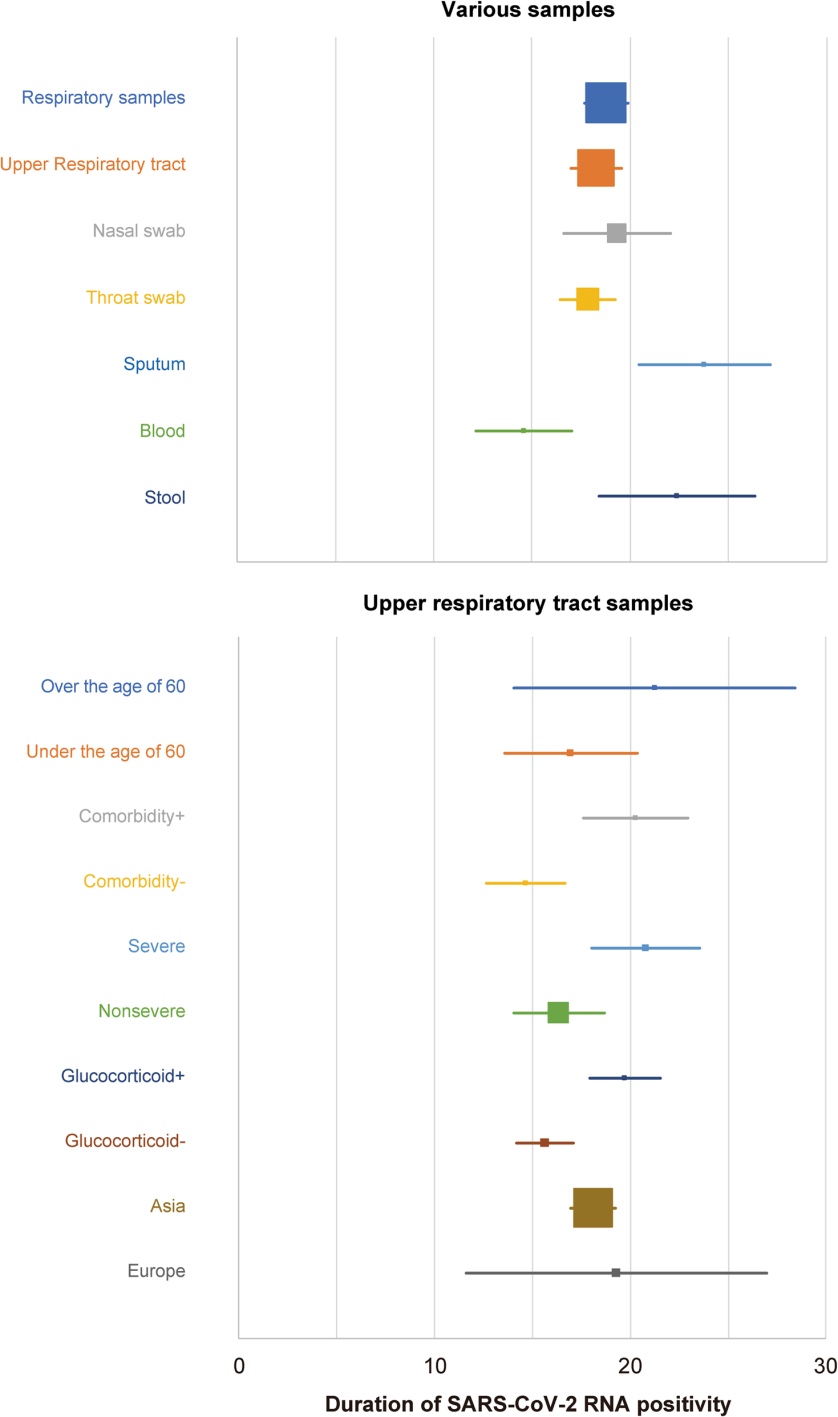
The summary of the duration of SARS-CoV-2 RNA positivity in the various samples and the clinical characteristics

The DSRP in various samples and various backgrounds are summarized in Fig. 10. An average of 18.29 days (95% CIs, 17.00–19.58 days) from the onset was required for the clearance of viral RNA from the upper respiratory tract samples. The DSRP on the sputum and the stool tended to be longer and that on the blood tended to be shorter. Due to analytical power, direct comparison showed that the DSRP was significantly longer than the upper respiratory tract samples in the stool alone.

The DSRP in the upper respiratory tract samples tended to be longer in patients older, with any comorbidities, severer, and treated with glucocorticoid, while it was not affected by gender and locality. The presence or absence of complications had the greatest impact on the difference in DSRP, although the effects of confounding factors cannot be ruled out.

## Discussion

The DSRP in the sputum tended to be longer than that in the upper respiratory tract. In the early phase of COVID-19, the Ct value of the RT-PCR in the sputum tended to be lower than that in the upper respiratory tract [80, 98]. The high viral load of SARS-CoV-2 in the lungs may be one of the reasons for the long DSRP in the sputum. The shorter DSRP in the blood than that in the upper respiratory tract may be due to the lower viral load of SARS-CoV-2 in the blood in the early phase of COVID-19 [7, 72]. On the other hand, the viral load in the stool in the early phase of COVID-19 was not much different from that in the upper respiratory tract [6, 7]. SARS-CoV-2 may avoid elimination by unknown mechanisms and continue to replicate in the gastrointestinal tract [112].

The age of patients may affect the DSRP in the upper respiratory tract based on the sensitivity analysis and the subgroup analysis. No reports of differences in Ct values of RT-PCR between the older and younger groups were found, but the peak viral load in saliva exhibited a positive correlation with age [113]. Aging led to a delay or dysfunction in the initial triggering of the immune response [114]. In addition, older patients are likely to have other factors that prolong DSRP. For example, the older people are likely to have comorbidities than younger people. The age has been reported as one of the risk factors for severe COVID-19 [115], and the activity of daily living was associated with prognosis in older patients with COVID-19 [116]. Although the effects of confounding factors could not be ruled out in this analysis due to many missing data values, the information that DSRP tends to be longer in the older patients is considered clinically useful.

The presence of any comorbidities may affect the DSRP in the upper respiratory tract based on the sensitivity analysis and the subgroup analysis. Ct values of RT-PCR in the patients with comorbidities were lower [86]. Hypertension, cardiovascular diseases, diabetes, and obesity related to abnormal immune response [117]. The outcomes of COVID-19 are primarily influenced by comorbidities and particular disease states or treatments in patients with rheumatic diseases [118]. In this analysis, it was not possible to analyze which diseases had an impact on the DSRP, and the effects of confounding factors could not be ruled out. However, the difference in the DSRP was the largest in the comparison between the patients with any complications and the patients without any complications.

The severity of COVID-19 may affect the DSRP in the upper respiratory tract. The viral load of SARS-CoV-2 was possibly high in patients with critically severe COVID-19 [113]. The reduction of viral load correlated with the seroconversion in SARS [116] and the seroconversion was delayed in patients with severe COVID-19 [119]. It was reported that the period from the first confirmation of SARS-CoV-2 to the confirmation of clearance was 10 days in asymptomatic patients, which was shorter than 16 days in symptomatic patients [92]. In the subgroup analysis with a uniform patient background, the effect of severity on DSRP was mild, but the presence or absence of symptoms and severity definitely affect DSRP.

The usage of glucocorticoid may affect the DSRP in the upper respiratory tract. Initially, glucocorticoids were basically deprecated because they seemed to worsen viral clearance based on SARS [120]. As expected, DSRP tended to be longer in the patients treated with glucocorticoid in the subgroup analysis. However, the use of dexamethasone resulted in lower 28-day mortality among patients with severe COVID-19 [121]. Glucocorticoids should be used in severe patients because of delayed virus clearance.

### Limitations

This study had several limitations. First, the positive result of the RT-PCR test does not always indicate the existence of transmittable SARS-CoV-2. Second, patients whose RT-PCR result for SARS-CoV-2 did not turn negative during the observation period were excluded. This study may underestimate the DSRP. Third, the funnel plots suggested the presence of bias or systemic heterogeneity. Fourth, the patient backgrounds in selected studies could not be fully unified. This may be a cause of the relatively high heterogeneity. It was difficult to reduce the heterogeneity enough with subgroup analyses. It may be possible to reduce heterogeneity if a more detailed patient background is available. Fifth, there were too many missing values. Multiple regression analysis could not be performed in the sensitivity analysis, and the number of patients was too small to further adjust the patient background in some subgroup analyses. In addition, it was not possible to assess which complications most affected the DSRP. Sixth, the effects of other drugs except for glucocorticoids on the DSRP could not be evaluated due to the small number of studies. Finally, the observational period of the included studies was until Jun 2020. The impact of SARS-CoV-2 variants, new therapies, and vaccinations on the DSRP could not be assessed.

## Conclusion

We summarized the duration of SARS-CoV-2 RNA positivity from various specimens and clinical characteristics in patients with COVID-19. The DSRP in the upper respiratory tract samples was 18.29 days, and the DSRP in the sputum and stool samples tended to be longer. Age, comorbidity, severity, and usage of glucocorticoid possibly affected the DSRP. Our results provide the basic data for the natural course of COVID-19 and may be especially useful information for people at risk of severe COVID-19. In the future, the impact of vaccination against SARS-CoV-2 and the SARS-CoV-2 variants on the duration of RNA positivity and comparison between RT-PCR and other methods such as antigen test should be assessed.

## Data Availability

The data underlying this article are available in the article.

## Abbreviations

CIs: Confidence intervals
COVID-19: Coronavirus disease 2019
Ct: Threshold cycle
DSRP: Duration of SARS-CoV-2 RNA positivity
RT-PCR: Reverse transcription-polymerase chain reaction
RNA: Ribonucleic acid
SARS-CoV-2: Severe acute respiratory syndrome coronavirus 2
SD: Standard deviation
